# Remote Sensing Image Recognition Based on LOG-T-SSA-LSSVM and AE-ELM Network

**DOI:** 10.1155/2022/8077563

**Published:** 2022-01-25

**Authors:** Chang-Jian Sun, Fang Gao

**Affiliations:** ^1^College of Electronic Science and Engineering, Jilin University, Changchun 130012, China; ^2^College of Computer Science and Technology, Jilin University, Changchun 130012, China; ^3^Chang Guang Satellite Technology Co., Ltd., Changchun 130000, China

## Abstract

Aiming at the influence of different working conditions on recognition accuracy in remote sensing image recognition, this paper adopts hierarchical strategy to construct a network. Firstly, in order to establish the classification relationship between different samples, labeled samples are used for classification. A Logistic-T-distribution-Sparrow Search Algorithm-Least Squares Support Vector Machines (LOG-T-SSA-LSSVM) classification network is proposed. LOG-T-SSA algorithm is used to optimize parameters in LSSVM to establish a better network to achieve accurate classification between sample sets and then identify according to different categories. Through UCI dataset test, the accuracy of LOG-T-SSA-LSSVM network classification is significantly improved compared with that of contrast network. The autoencoder is integrated with Extreme Learning Machine, and the autoencoder is used to realize data compression. The advantages of Extreme Learning Machine (ELM) network, such as less training parameters, fast learning speed, and strong generalization ability, are fully utilized to realize efficient and supervised recognition. Experiments verify that the autoencoder-extreme learning machine (AE-ELM) network has a good recognition effect when the sigmoid activation function is selected and the number of hidden layer neurons are 2000. Finally, after image recognition under different working conditions, it is proved that the recognition accuracy of AE-ELM based on LOG-T-SSA-LSSVM classification is significantly improved compared with traditional ELM network and Particle Swarm Optimization-Extreme Learning Machine (PSO-ELM) network.

## 1. Introduction

As the basis of computer vision application, object detection is one of the most widely concerned problems in real life. Generally speaking, the universal target recognition mainly has two subtasks: one is to judge the class probability of a specific target, and the other is to give the specific location of the target. Target recognition algorithm plays a very important role in daily life and has been successfully used in facial recognition [1–3], pedestrian detection [4–7], video analysis [8–10], and beacon positioning and recognition [11, 12]. With the continuous development of machine learning and its continuous application in the field of target detection, the accuracy of target detection in common scenarios has been greatly improved. However, it is still a hot research issue for target detection in complex environments with a large number of targets and variable scales.

Existing target detection methods can be divided into two categories: methods based on manual feature construction and methods based on deep learning. The focus of the manual method is to extract the hand-made features to represent the temporal and spatial features of the video sequence [13, 14]. For instance, literature [15] proposed a new descriptor of spatial and temporal features based on optical flow information, called histograms of optical flow orientation and magnitude and entropy. Literature [16] shows through experiments that the histogram-oriented gradient (HOG) descriptor of grid is significantly better than the existing feature set. Literature [17] proposed a scheme based on support vector machines. The flexible genre model (FGM) is proposed [18], which aims to characterize the data population at the point level and population level to detect various types of population anomalies. Although the method based on manual feature construction has achieved some achievements, the traditional target detection algorithm based on manual feature construction is not suitable for solving existing problems because of its complicated process and large amount of calculation [19]. Recently, with the continuous success of deep learning technology in various fields, object detection based on deep learning has become a research hotspot. AlexNet [20], proposed in 2012, is the first deep neural network that has made a breakthrough in large-scale image recognition. After this, deep neural networks began to be widely used in the field of computer vision. For example, VGGNET was proposed in 2014 [21]. ResNet was proposed in 2016 [22]. ResNeXt was proposed in 2017 [23]. SENet [24] was proposed in 2018. ExtremeNet was proposed in 2019 [25].

Target detection is also a hot topic in remote sensing field. However, it should be noted that methods in the field of computer vision cannot be directly applied in the field of remote sensing [26, 27], because commonly used remote sensing images and natural images are quite different. For example, remote sensing images often capture the top features of the target, while natural images capture the contour features of the target. However, as deep learning-based methods have made great achievements in the field of target detection, related extended methods have also been applied to remote sensing images. Deep learning-based target detection methods can generally be divided into two categories: region proposal-based methods, namely, two-stage detection, and regression-based methods, represented as one-stage detection.

Two-stage detection divides the detection task into two stages: (1) proposal generation and (2) proposal prediction. The first phase focuses on generating a series of candidate region proposals that might contain objects. The objective of the second phase is to classify the candidate area proposals from the first phase into object classes or backgrounds and further fine-tune the coordinates of the bounding boxes. In the two-stage algorithm, the representative method is R-CNN [28] as well as the variant method based on R-CNN, such as Faster R-CNN [29] and rotation-invariant CNN [30].

Although R-CNN and its variant methods have been successfully applied in the field of remote sensing image detection, it is undeniable that the training process is very clumsy and slow. Recently, in order to achieve real-time target detection, some researchers have begun to study the detection method based on regression, also known as one-stage detection. For example, Tang proposed Oriented_SSD (Single Shot MultiBox Detector), which improved the efficiency and accuracy of vehicle detection [31, 32]. Liu proposed SSD and its validity was verified through multiple datasets [33–35].

In this paper, a hierarchical strategy is used to construct a network for remote sensing image recognition. Firstly, in order to establish the classification relationship between different samples, labeled samples are used for classification. A LOG-T-SSA-LSSVM classification network is proposed. LOG-T-SSA algorithm is used to optimize parameters in LSSVM to establish a better network to achieve accurate classification between sample sets and then identify according to different categories. The autoencoder is integrated with Extreme Learning Machine, and the autoencoder is used to realize data compression. The advantages of ELM network, such as less training parameters, fast learning speed, and strong generalization ability, are fully utilized to realize efficient and supervised recognition.

The rest of this article is arranged as follows. The second part introduces LOG-T-SSA-LSSVM classifier. The third part introduces AE-ELM network recognizer. The fourth part constructs the recognizer combining LOG-T-SSA-LSSVM and AE-ELM. The fifth part carries on the relevant experiment verification. The last part is the conclusion and future development.

## 2. LOG-T-SSA-LSSVM Classifier and AE-ELM Recognizer

### 2.1. The LOG-T-SSA Algorithm

Sparrow Search Algorithm (SSA) is a new swarm intelligence optimization algorithm [36], which is superior to Grey Wolf Optimizer (GWO), Particle Swarm Optimization (PSO), Gravity Search Algorithm (GSA), and other algorithms. Entrants, scouts, and discoverers are mainly responsible for guiding the overall optimization direction of the population. Compared with entrants, their search scope is larger, generally accounting for 10% to 20% of the total population. The sparrows with better performance in each iteration take the role, and their specific position update formula is as follows:

(1) $$\begin{array}{c} x_{i,j}^{t+1}=\left\{\frac{x_{i,j}^{t}\cdot e^{\left(-i/\left(\alpha \cdot iter\_max\right)\right)}R<\text{ST}}{x_{i,j}^{t}+Q\cdot \text{LR}\geq \text{ST}}\right), \end{array}$$

where sparrow is in row *i* and column *j* in generation *t*+1 of *x*_*i*,*j*_^*t*+1^. *t* represents the current iteration number. *α* represents random number with a range of [0, 1]. Warning value *R* is in the range of [0, 1]. Safe value ST is in the range of [0.5, 1]. *Q* is a random number subject to standard integer normal distribution. *L* is a matrix of 1 × *D*, where *D* is the latitude of the problem, and all elements are 1.

The entrants are nondiscoverers, and the proportion of them always remains the same. The updating formula is related to discoverers, and the formula is as follows:

(2) $$\begin{array}{c} x_{i,j}^{t+1}=\left\{\frac{x_{\text{best}}^{t}+\beta \left|x_{i,j}^{t}-x_{\text{best}}^{t+1}\right|f_{i}>f_{g\_\text{best}}}{x_{i,j}^{t}+k\left(\left|x_{i,j}^{t}-x_{\text{worst}}^{t}\right|/f_{i}-f_{g\_\text{worst}}+\xi \right)f_{i}=f_{g\_\text{best}}}\right), \end{array}$$

where *x*_best_^*t*^ represents the individual with the worst fitness value in the iteration of *t* generation and *x*_*p*_^*t*+1^ represents the individual with the best fitness value in the iteration of *t*+1 generation. *A* is the matrix of 1 × *D*. The elements are either 1 or −1. *A*^+^=*A*^*T*^(*AA*^*T*^)^−1^.

The scout is jointly held by the discoverer and the entrant, indicating that the dangerous individuals are aware of in the population, accounting for 10%∼20% of the total population. The position update formula is as follows:

(3) $$\begin{array}{c} x_{i,j}^{t+1}=\left\{\frac{x_{\text{best}}^{t}+\beta \left|x_{i,j}^{t}-x_{\text{best}}^{t+1}\right|f_{i}>f_{g\_\text{best}}}{x_{i,j}^{t}+k\left(\left|x_{i,j}^{t}-x_{\text{worst}}^{t}\right|/f_{i}-f_{g\_\text{worst}}+\xi \right)f_{i}=f_{g\_\text{best}}}\right), \end{array}$$

where *x*_best_^*t*^ represents the population individual with the best fitness in generation *t*. *β* follows the standard normal distribution and controls the update step size. *k* belongs to [−1,1] and is a random number. *f*_*i*_ represents the current individual fitness value. *f*_*g*_best_ represents the current global optimal fitness. *f*_*g*_worst_ represents the current worst fitness value, and *ξ* is a constant term to prevent the denominator from being 0.

In order to improve the initial population quality of the algorithm, logistic mapping is introduced [37]. To a certain extent, logistic mapping is a time-discrete demographic model, which can fully demonstrate chaos dynamics. The expression is as follows:

(4) $$\begin{array}{c} x\left(t\right)=\mu \cdot x\left(t\right)\cdot \left[1-x\left(t\right)\right], \end{array}$$

where *t* is the number of iterative steps; *x*(*t*) ∈ [0,1]. *μ* is the adjusting parameter. To ensure that the mapping range is between 0 and 1, *μ* ∈ [0,4]. *x*(*t*) is the proportion of the population to the maximum possible population size at time *t* (i.e., the ratio of the existing population to the maximum possible population). When parameter *μ* is changed, the equation will show different dynamic limit behaviour. When 0 < *μ* < 1, the limit behaviour of the population tends to a fixed value of 0. When *μ* is between 1 and 3, the population value will approach (*μ* − 1)/*μ*. Different *μ* values can adjust the convergence rate. When *μ* is between 1 and 4, it will show periodic fluctuations. At the same time, the adaptive T-distribution is introduced to improve the update step size, and the T-distribution update formula is as follows:

(5) $$\begin{array}{c} x_{i}^{t+1}=x_{i}^{t}+x_{i}^{t}\cdot t\left(\text{iter}\right), \end{array}$$

where *x*_*i*_^*t*+1^ is the position of sparrow after mutation. *x*_*i*_^*t*^ is the position of the ith individual sparrow of *t* generation. *t*(iter) is the T-distribution of the degree of freedom taking the number of iterations of the algorithm as the parameter. This formula makes full use of the current population information and takes the number of iterations *t* as the degree of freedom parameter. Cauchy-like variation with small *t* in the early stage has strong global search ability, and gauss variation with large *t* in the late stage has strong local search ability. Thus, the search ability of the algorithm is improved.

### 2.2. LSSVM Network

Least Squares Support Vector Machine (LSSVM) is a method to transform support vector machine into linear problem [38]. Through the sum of squares of minimum error, the fitting object is close to the target. LSSVM changed the inequality constraint in SVM to equality constraint, and the LSSVM structural risk minimization formula is as follows:

(6) $$\begin{array}{c} \text{min}J\left(w,e\right)=\frac{1}{2}w^{T}w+\frac{\gamma }{2}\sum_{K=1}^{N}e_{k}^{2}, \end{array}$$

where min*J*(*w*, *e*) is the objective function, *w* is the weight coefficient, *γ* is the penalty factor, and *e* is the error; the equation condition is as follows:

(7) $$\begin{array}{c} y_{i}w^{T}\varphi \left(x_{i}\right)be_{i}=0, \end{array}$$

where *y*_*i*_ is the corresponding output variable. *φ*(*x*_*i*_) is the nonlinear transformation function of input data. **w** is the weight vector. *b* is the bias term. Lagrange function is constructed by the following formula:

(8) $$\begin{array}{c} L\left(w,b,e,\alpha \right)=J\left(w,e\right)-\sum_{i}^{N}\alpha_{i}\left[\left(w^{T}\varphi \left(x_{i}\right)+b\right)+e_{i}-y_{i}\right]\\ =\frac{1}{2}w^{T}w+\frac{\gamma }{2}\sum_{i}^{N}e_{i}^{2}-\sum_{i}^{N}\alpha_{i}\left[\left(w^{T}\varphi \left(x_{i}\right)+b\right)+e_{i}-y_{i}\right], \end{array}$$

where *L*(**w**, *b*, *e*, *α*) is the Lagrange expression and *α* is the Lagrange multiplier. According to the Karush-Kuhn-Tucker (KKT) optimization conditions, the following conditions are satisfied:

(9) $$\begin{array}{c} \left\{\begin{array}{c} \frac{\partial L}{\partial w}=0,\\ \frac{\partial L}{\partial b}=0,\\ \frac{\partial L}{\partial e}=0,\\ \frac{\partial L}{\partial \alpha }=0. \end{array}\right) \end{array}$$

### 2.3. Optimizing the LSSVM Network

**Figure 1:**
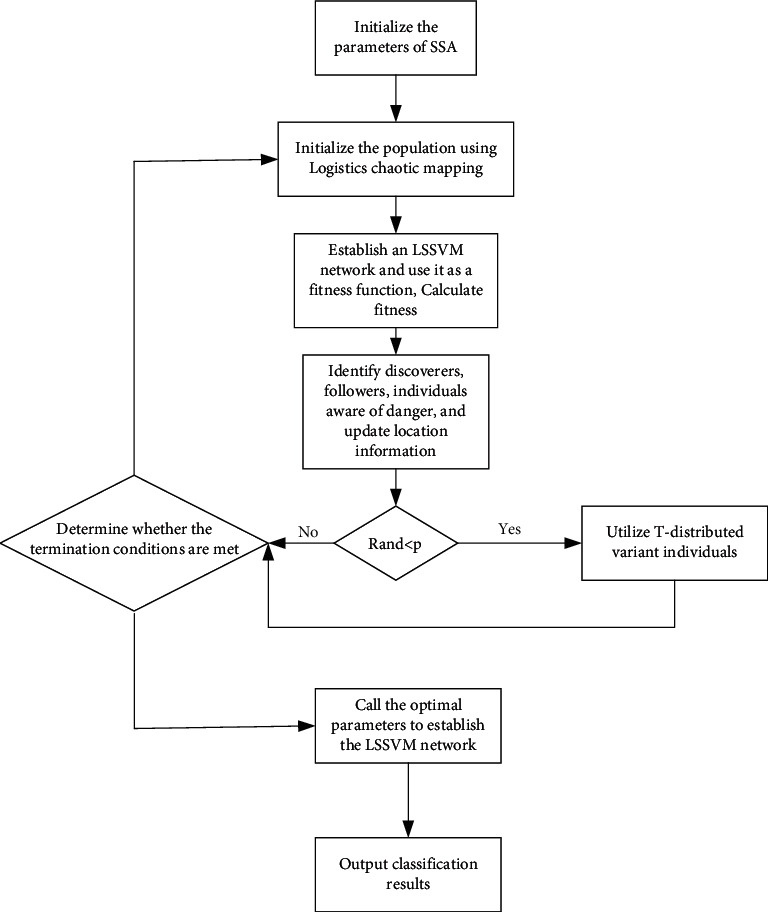
Flowchart of optimizing LSSVM by LOG-T-SSA algorithm.

LOG-T-SSA algorithm was used to optimize LSSVM parameters. The specific process is shown in Figure 1, and the steps are as follows:

  Step 1: initialize the parameters of the sparrow search algorithm, including the initial number of sparrow population *n*, the proportions of finder and follower in the population, the warning value *R*, the safety value ST, the random value *Q*, and other parameters
  Step 2: use logistic chaos mapping function to generate chaotic sequence, that is, individual member of sparrow population in the initial solution space position
  Step 3: establish LSSVM network, and take LSSVM network classification error rate as fitness function
  Step 4: calculate the fitness value of each sparrow to determine the individual position of the optimal solution and the worst solution
  Step 5: identify the finder in the population and update the location of the finder
  Step 6: identify the follower and update the position of the follower
  Step 7: determine the number of dangerous individuals in the population and calculate the update position
  Step 8: *R* and < *p*, T-distribution variation for individuals
  Step 9: calculate the population fitness before and after variation and determine the optimal solution of the population
  Step 10: if the maximum number of iterations is reached or the threshold is met, output the optimal kernel parameter and penalty factor; if it is met, go back to Step 4
  Step 11: LSSVM network was established by using optimal kernel parameters and penalty factors, classification was carried out, and classification results were output

### 2.4. Optimizing the LSSVM Network

In order to test the classification ability of LOG-T-SSA-LSSVM network, glass dataset in UCI was selected for verification. Three comparative classification networks were selected, namely, SSA-LSSVM, Tent-SSA-LSSVM, and EOBL-SSA-LSSVM. The experimental simulation environment was Windows 10, CPU: 2.80 GHz, 16 GB memory, operating environment: Matlab 2019b. The classification network parameters are shown in Table 1.

**Figure 2:**
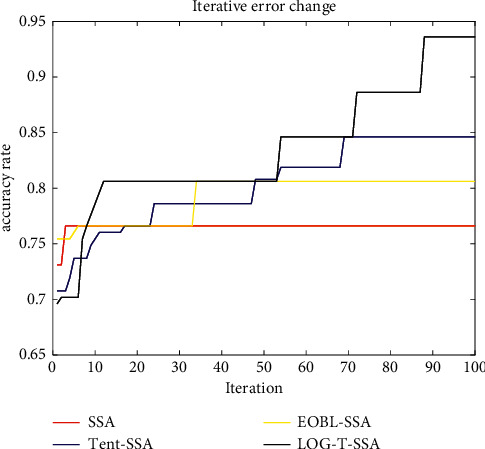
Iteration curve of accuracy.

In Figure 2, accuracy is used as the evaluation standard. Under the same number of iterations, it can be seen intuitively that LOG-T-SSA algorithm has a faster convergence speed in the initial stage, indicating that logistic chaotic mapping enables the population to have a good initial distribution and the population diversity increases significantly. In the process of population renewal, compared with SSA, Tent-SSA, and EOBL-SSA, SSA had stronger optimization performance. SSA fell into local optimum within 10 generations, EOBL-SSA fell into local optimum within 40 generations, and Tent-SSA fell into optimum within 70 generations. However, LOG-T-SSA does not stop optimization until less than 90 generations, indicating that it has better search performance.

**Figure 3:**
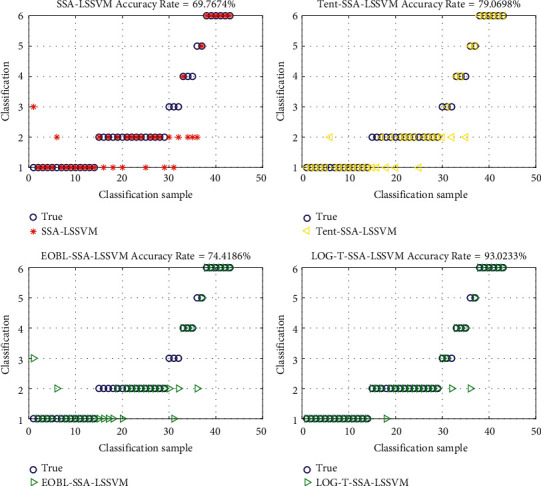
Classification results of glass datasets.

Figure 3 shows that, for glass datasets, the classification accuracy of SSA-LSSVM is 69.7%, that of Tent-SSA-LSSVM is 79.0%, and that of EOBL-SSA-LSSVM is 74.4%. By contrast, the classification accuracy of LOG-T-SSA network is as high as 93%. It shows that the improved classification network in this paper has good classification ability on the multicategory dataset.


(10) $$\begin{array}{c} \left\{\begin{array}{c} \dot{x}=v\text{cos}\gamma \text{cos}\psi ,\\ \dot{y}\;=v\text{cos}\gamma \text{sin}\psi ,\\ \dot{h}\;=v\text{sin}\gamma ,\\ \dot{v}=g\left(n_{x}-\text{sin}\gamma \right),\\ \dot{\gamma }=\frac{g}{v}\left(n_{z}\text{cos}\mu -\text{cos}\gamma \right),\\ \dot{\chi }=\frac{g}{v\text{cos}\gamma }n_{z}\text{sin}u. \end{array}\right) \end{array}$$

## 3. AE-ELM Network

### 3.1. Autoencoder

**Figure 4:**
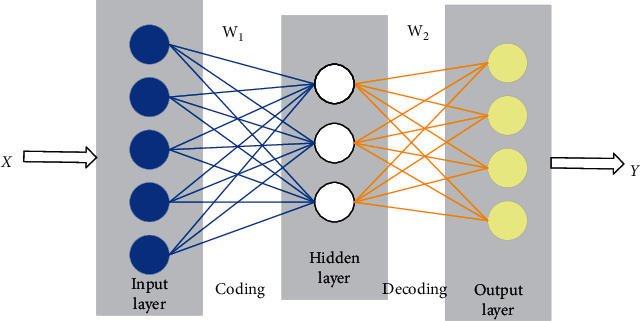
Network structure diagram of autoencoder.

Autoencoder is an artificial neural network for unsupervised learning, which consists of three layers: input layer, output layer, and hidden layer [39]. At present, there are two main applications of autoencoders, one is data denoising, and the other is for visual and dimension reduction. Since high-dimensional data are often located in a low-dimensional manifold or nearby, the encoder nonlinearly maps the input data set to the hidden layer through the encoding process, and the data set is compressed and encoded. That is, the characteristic information of the original data in another dimension space can be obtained, which is enough to reproduce the information of the input layer, so as to achieve the purpose of reducing the data dimension and improving the computing efficiency. The network structure of autoencoder is shown in Figure 4.

Autoencoder (AE) consists of encoding and decoding. The encoding process is to map input *x* to the hidden layer through a nonlinear activation function. The decoding process is to transform the hidden layer data *h* into the output value *Y* to reconstruct the input. Encoding process formula is

(11) $$\begin{array}{c} h=f\left(x\right)=s_{f}\left(W_{1}x+b\right). \end{array}$$

The decoding formula is

(12) $$\begin{array}{c} Y=g\left(h\right)=s_{g}\left(W_{2}x+\tilde{b}\right). \end{array}$$

The loss function is

(13) $$\begin{array}{c} J_{AE}\left(\theta \right)=\frac{1}{N}\sum_{i=1}^{N}L\left(X_{i},Y_{i}\right), \end{array}$$

where *s* is the activation function. *W*_1_ and *b* are the encoder weight and bias, respectively. *W*_2_ and $\tilde{b}$ are the decoder weight and bias, respectively. *L*(*x*_*i*_, *y*_*i*_)=1/2‖*x*_*i*_ − *y*_*i*_‖^2^ is the error function.

### 3.2. ELM Network

**Figure 5:**
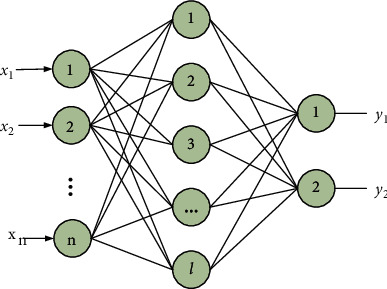
ELM network structure diagram.

Extreme Learning Machine (ELM) is a single hidden layer feedforward neural network [40]. The network structure model of ELM is shown in Figure 5. The training set has *N* samples {*x*, *y*}={(*x*_*i*_, *y*_*i*_)*|x*_*i*_ ∈ *R*^*dn*^, *y*_*i*_ ∈ *R*, *i*=1,2...*N*}. Then its model is

(14) $$\begin{array}{c} f\left(x_{i}\right)=\beta g\left(Wx_{i}+b\right), \end{array}$$

where *W* is the weight vector from the input layer to the hidden layer. *b* is the bias vector. *β* is the output weight from the hidden layer to the output layer. ELM matrix expression is

(15) $$\begin{array}{c} H\beta =Y, \end{array}$$

where **H** is the output matrix of the hidden layer and **Y** is the real matrix of the sample target output. The training process is to solve the least squares solution *β*; namely,

(16) $$\begin{array}{c} \text{min}\left(H\beta -Y\right). \end{array}$$

The output weight matrix can be solved by Moore-Penrose generalized inverse formula to obtain

(17) $$\begin{array}{c} \beta =H^{+}Y. \end{array}$$

**H** ^+^=(**H**^**T**^**H**)^−1^**H**^**T**^ [41].

### 3.3. AE-ELM Network Settings

After AE data dimensionality reduction, ELM network is used for fast recognition. For ELM network, different activation functions have different recognition effects. In order to select the most suitable activation function, Root Mean Square Error was used as an evaluation index, and the optimal activation function was selected through iterative calculation of different neuron numbers.

**Figure 6:**
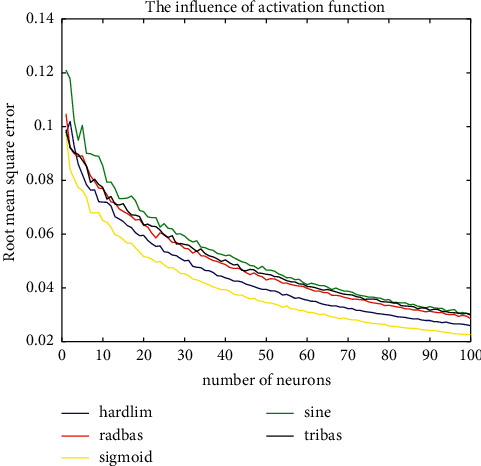
Activation function selection.

Hardlim function, Radbas function, Sigmoid function, Sine function, and Tribas function are selected, respectively, and Inria Aerial Image Labeling dataset is used for training. Root Mean Square Error (RMSE) results are shown in Figure 6. It can be seen intuitively that Sigmoid function has the best activation effect. The Sine function has the worst effect. There is no significant difference between Hardlim, Radbas, and Tribas functions. Therefore, this article selects the Sigmoid function as the activation function.

**Figure 7:**
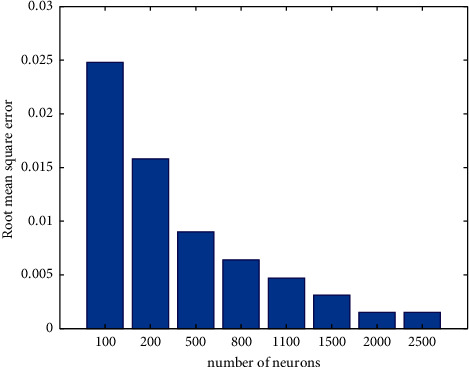
Selection of number of neurons.

The number of hidden layer neurons in ELM network has significant influence on the recognition result. In order to select the optimal structure, on the basis of determining the activation function, different numbers of neurons are set for recognition, and the recognition results are shown in Figure 7. It can be seen that, with the increase of the number of hidden layer neurons, the recognition effect of the network is also significantly improved. However, when the number of neurons reaches 2000, the Root Mean Square Error (RMSE) cannot be significantly reduced by increasing the number of neurons, so the number of network neurons in this paper is 2000.

## 4. Constructing a Recognizer Combined with LOG-T-SSA-LSSVM and AE-ELM

**Figure 8:**
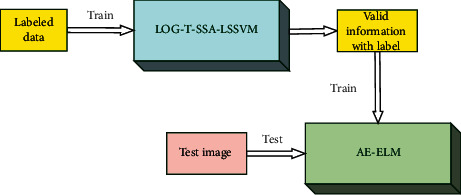
Flowchart of LOG-T-SSA-LSSVM combined with AE-ELM.

In the process of image shooting, angle is not uniform; it is a major problem. In this paper, the LOG-T-SSA-LSSVM network is used to fit the relationship between images and labels, and a strong classification network is established to extract effective information. On this basis, AE-ELM network is used to compress and extract data, and supervised learning method is used to establish a high accuracy recognizer. The process is shown in Figure 8, and the steps are as follows:

  Step 1: train the LOG-T-SSA-LSSVM network with labeled data
  Step 2: input images into the LOG-T-SSA-LSSVM network after training to extract effective information
  Step 3: input effective information and labels as AE-ELM network to train AE-ELM network
  Step 4: use the test image to verify the recognition accuracy

## 5. Experimental Verification

In order to verify the recognition accuracy of the method proposed in this paper, image sets under three different working conditions were selected. The recognition sample data were all extracted from the WIDE-amplitude star L2E class data product of JL-1. After orthofusing-drop processing, they were RGB true color 8-bit image products with a resolution of 0.75 m and taken on October 18, 2020. The shooting location is the main city of Changchun.

The JL101K satellite can obtain high-resolution panchromatic images and multispectral images. The pendulum angle of the imaging can be customized according to user requirements and is widely used in economic survey, disaster prevention and mitigation, social development research, and other fields. The main indicators are shown in Table 2.

The traditional ELM network and PSO-ELM network were selected as the comparison recognizers, and the recognition was carried out under three working conditions. The experimental simulation environment was Windows 10, and CPU was 2.80 GHz, with 16 GB memory, and the operating environment was Matlab 2019b. Network parameters are shown in Table 3.

**Figure 9:**
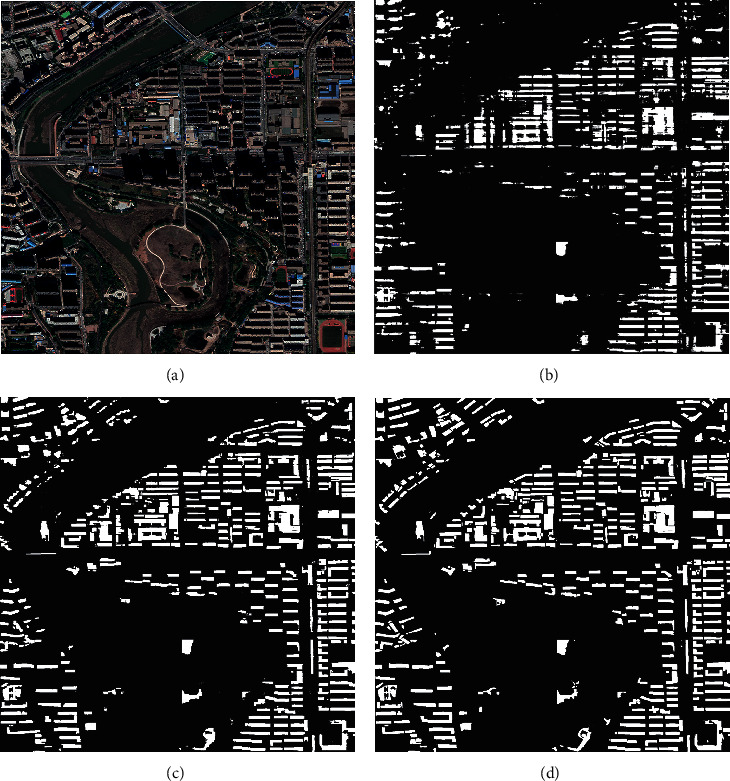
Recognition results of Case 1. (a) The original image. (b) ELM recognition result. (c) PSO-ELM recognition result. (d) AE-ELM recognition result.

As can be seen from Figure 9, the ELM network recognition is fuzzy. Small individuals cannot be recognized, and they are vulnerable to the influence of edge signals. Compared with ELM network, PSO-ELM network can better identify objects through algorithm optimization, but its recognition accuracy also decreases significantly in complex geographical situations. After the image is processed by LOG-T-SSA-LSSVM classification, the output to AE-ELM network can achieve a better recognition effect.  Table 4 shows that its recognition accuracy is as high as 99.11%, which has been significantly improved.

**Figure 10:**
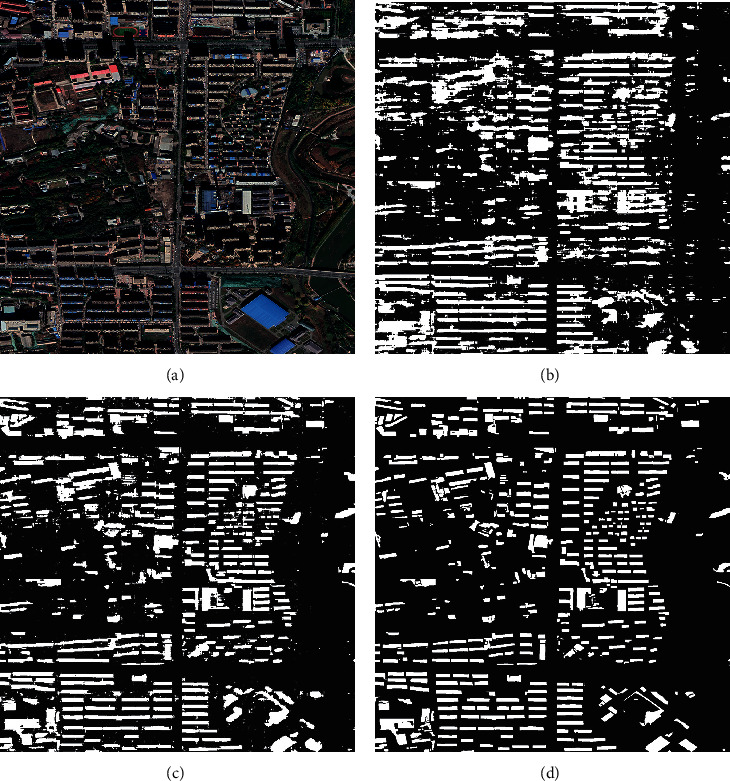
Case 2 recognition results. (a) The original image. (b) ELM recognition result. (c) PSO-ELM recognition result. (d) AE-ELM recognition result.

As can be seen from Figure 10, the recognition accuracy of ELM and PSO-ELM has decreased significantly, and the recognition accuracy is below 80%. The poor processing ability of images from different angles indicates that the network universality is poor without LOG-T-SSA-LSSVM first classification. Although the recognition accuracy of AE-ELM network decreased slightly, it can be seen from Table 4 that the accuracy still remains above 90%. Therefore, LOG-T-SSA-LSSVM was first used to classify the sampled images, and then images of different categories were identified, and the accuracy was significantly improved.

## 6. Conclusion

In this paper, a hierarchical strategy is used to construct a network for remote sensing image recognition. Firstly, in order to establish the classification relationship between different samples, labeled samples are used for classification. A LOG-T-SSA-LSSVM classification network is proposed. LOG-T-SSA algorithm is used to optimize parameters in LSSVM to establish a better network to achieve accurate classification between sample sets and then identify according to different categories. The autoencoder is integrated with Extreme Learning Machine, and the autoencoder is used to realize data compression. The advantages of ELM network, such as fewer training parameters, fast learning speed, and strong generalization ability, are fully utilized to realize efficient and supervised identification. The following conclusions are drawn after the test verification:

Through UCI dataset test, LOG-T-SSA-LSSVM network classification has significantly improved classification accuracy compared with SSA-LSSVM, Tent-SSA-LSSVM, and EOBL-SSA-LSSVM.

After image recognition under different working conditions, the recognition accuracy of AE-ELM based on LOG-T-SSA-LSSVM classification is significantly improved compared with traditional ELM network and PSO-ELM network.

The future research direction will focus on image recognition in fuzzy background.

## Figures and Tables

**Table 1:** Comparison algorithm parameter table.

Algorithm	Parameter setting
SSA-LSSVM	ST = 0.6; PD = 0.4; initial kernel parameter 20; initial penalty factor 100
Tent-SSA-LSSVM	Tent Beta = 0.4; ST = 0.6; PD = 0.4; initial kernel parameter 20; initial penalty factor 100
EOBL-SSA-LSSVM	Learning rate 0.5; ST = 0.6; PD = 0.4; initial kernel parameter 20; initial penalty factor 100
LOG-T-SSA-LSSVM	Log coefficient 0.4; T-distribution degree of freedom is the number of iterations; ST = 0.6; PD = 0.4; initial kernel parameter 20; initial penalty factor 100

**Table 2:** Test data information.

Technical index	Parameters
Ground pixel resolution of subsatellite point	0.75 m (panchromatic)/3 m (multispectral)
Parrot sequoia	(a) Panchromatic P: 450 nm to 800 nm
	(b) Blue B1: 450 nm to 510 nm
	(c) Green B2: 510 nm to 580 nm
	(d) Red B3: 630 nm to 690 nm
	(e) Simulation near-infrared B4: 770 nm to 895 nm
Digitalizing bit	12 bits
Standard scene size (substellar point)	23 km × 23 km (6-point camera mode, default)
	46 km × 46 km (3-point camera mode)
Orbit altitude	481.56 km
Positioning accuracy without control (CE90)	20 m

**Table 3:** Comparison network parameters.

Algorithm	Parameter setting
ELM	Number of neurons: 2000
PSO-ELM	c1 = 2; c2 = 2; maximum number of iterations: 1000; number of neurons: 2000
AE-ELM	Number of AE layers: 15; number of ELM neurons: 2000

**Table 4:** RMSE identification and accuracy.

	Case 1 (RMSE/accuracy)	Case 2 (RMSE/accuracy)
ELM	0.0603/92.93%	0.1178/69.58%
PSO-ELM	0.0414/97.44%	0.0782/75.28%
AE-ELM	0.0018/99.11%	0.0025/93.32%

## Data Availability

The data used to support the findings of this study are included within the article.
